# Safety of Benzathine Penicillin for Preventing Congenital Syphilis: A Systematic Review

**DOI:** 10.1371/journal.pone.0056463

**Published:** 2013-02-21

**Authors:** Tais F. Galvao, Marcus T. Silva, Suzanne J. Serruya, Lori M. Newman, Jeffrey D. Klausner, Mauricio G. Pereira, Ricardo Fescina

**Affiliations:** 1 University of Brasilia, Faculty of Medicine, Brasilia, Distrito Federal, Brazil; 2 Pan-American Health Organization, Centro Latinoamericano de Perinatología/Salud de la Mujer y Reproductiva (CLAP/SMR), Montevideo, Uruguay; 3 Department of Reproductive Health and Research, World Health Organization, Geneva, Switzerland; 4 Division of Infectious Diseases and Program in Global Health, David Geffen School of Medicine, University of California Los Angeles, Los Angeles, California, United States of America; University of Vermont College of Medicine, United States of America

## Abstract

**Objective:**

To estimate the risk of serious adverse reactions to benzathine penicillin in pregnant women for preventing congenital syphilis.

**Methods:**

We searched for clinical trials or cohorts that assessed the incidence of serious adverse reactions to benzathine penicillin in pregnant women and the general population (indirect evidence). MEDLINE, EMBASE, Scopus and other databases were searched up to December 2012. The GRADE approach was used to assess quality of evidence. Absolute risks of each study were calculated along with their 95% confidence intervals (95% CI). We employed the DerSimonian and Laird random effects model in the meta-analyses.

**Results:**

From 2,765 retrieved studies we included 13, representing 3,466,780 patients. The studies that included pregnant women were conducted to demonstrate the effectiveness of benzathine penicillin: no serious adverse reactions were reported among the 1,244 pregnant women included. In the general population, among 2,028,982 patients treated, 4 died from an adverse reaction. The pooled risk of death was virtually zero. Fifty-four cases of anaphylaxis were reported (pooled absolute risk = 0.002%; 95% CI: 0%–0.003% I^2^ = 12%). From that estimate, penicillin treatment would be expected to result in an incidence of 0 to 3 cases of anaphylaxis per 100,000 treated. Any adverse reactions were reported in 6,377 patients among 3,465,322 treated with penicillin (pooled absolute risk = 0.169%; 95% CI: 0.073%–0.265% I^2^ = 97%). The quality of evidence was very low.

**Conclusion:**

Studies that assessed the risk of serious adverse events due to benzathine penicillin treatment in pregnant women were scarce, but no reports of adverse reactions were found. The incidence of severe adverse outcomes was very low in the general population. The risk of treating pregnant women with benzathine penicillin to prevent congenital syphilis appears very low and does not outweigh its benefits. Further research is needed to improve the quality of evidence.

## Introduction

Over half of syphilis infections in pregnancy will result in congenital syphilis, which can manifest as early fetal loss, stillbirth, prematurity, low birth weight, neonatal death, or infection in the newborn [1]. Benzathine penicillin is the only effective treatment for preventing congenital syphilis [2], [3]. In contrast with other antimicrobials, the effectiveness of penicillin for syphilis and the sensitivity of *Treponema pallidum* to penicillin remains stable [4]. Since penicillin became available in 1943, the rates of congenital syphilis and deaths due to syphilis have dramatically decreased [4]. The use of penicillin in pregnant women with syphilis significantly reduces the risk of congenital syphilis, perinatal death, stillbirth and preterm delivery [5]–[9]. The global elimination of congenital syphilis is feasible and thus a major priority in public health [10].

Despite the widespread availability, low cost, and effectiveness of penicillin in controlling congenital syphilis, the worldwide incidence is still high [10], and the rates in some countries are increasing [11]–[15]. Annually more pregnancies are adversely affected by syphilis than by HIV infection [3]. Among the multiple causes of the inability to eliminate the mother-to-child transmission of syphilis are concerns about the safety of penicillin injections, mainly in resource-constrained settings and in patients with a history of penicillin allergy [4]. Anecdotal reports suggest that some rural or primary care health workers might not feel comfortable administering injectable penicillin.

In many settings, prior to penicillin benzathine administration, a subcutaneous or subdermal injection of benzathine penicillin is used as a screening method for an allergic reaction [16]. Hypersensitivity to penicillin, however, is often due to minor determinants and other metabolites of penicillin not contained in the parent molecule, which renders such tests limited in their ability to accurately predict whether patients will have an adverse reaction or not. Such testing might provide false positive or false negative results and thus misclassify patients [17]. A detailed clinical history accurately diagnoses hypersensitivity and helps in judging the clinical relevance of the symptoms, as some are not predictive or related to allergy [18]. Careful clinical examination should also be emphasized in patients with suspected penicillin allergy. It has been estimated that 90% of self-reported allergies are mislabeled, often because of confusion between adverse reactions and disease symptoms [19].

While speculations about penicillin hazards are frequent, systematic reviews about the incidence of serious adverse reactions in pregnancy are absent. Such evidence is likely to bring more objectivity to the clinical and policy decisions. Our aim was to review the risk of serious adverse reaction to benzathine penicillin in pregnant women with syphilis.

## Methods

### Protocol and Registration

The current review was registered on International Prospective Register of Systematic Reviews (PROSPERO), registration number: CRD42012002103.

### Eligibility Criteria

We included randomized controlled trials (RCT) or cohort studies that assessed the incidence of serious adverse reactions to benzathine penicillin in pregnant women for preventing congenital syphilis. As indirect evidence, we also included studies that assessed the incidence of adverse reactions to benzathine penicillin in the general population. There were no restrictions for language, length of follow-up, publication date or status.

We used the following adverse reaction definition “an appreciably harmful or unpleasant reaction, resulting from an intervention related to the use of a medicinal product, which predicts hazard from future administration and warrants prevention or specific treatment, or alteration of the dosage regimen, or withdrawal of the product” [20], which includes drug allergy and allergic anaphylaxis [21]. Non-allergic adverse reactions reported by authors such as diarrhea, nausea, and vomiting were also included. We did not consider Jarisch-Herxheimer reaction as an adverse reaction, as its physiopathologic mechanism is related to a reaction to *T. pallidum* proteins rather than to penicillin [22].

We only included studies that fulfilled all inclusion criteria. If the study did not provide a clear definition of adverse reactions, we reviewed the primary data and assessed which studies contained data that could be categorized using our criteria. Once a study was accepted for inclusion, no patients were reassigned into or out of adverse reaction groups. Results were taken as a group from each study, as individual patient-level reporting was not available.

### Information Sources and Search Strategy

We searched the following databases from inception up to January 2013: MEDLINE, EMBASE, Scopus, Cumulative Index to Nursing and Allied Health Literature (CINAHL), Reactions Pharmacovigilance Insight (via OVID), Cochrane Central Register of Controlled Trials (CENTRAL), metaRegister of Current Clinical Trials (mRCT), Latin American and Caribbean Center on Health Sciences Information (LILACS) and Scientific Electronic Library Online (SciELO). References of relevant studies were also screened for eligibility. The search strategy for MEDLINE (via PubMed) is presented on Table S1. This strategy was adapted for searching on the other databases.

### Study Selection

Two researchers independently reviewed the retrieved studies based on the analysis of the titles and abstracts (MCM and MCRS). Disagreements were resolved by authors’ consensus or by a third reviewer (TFG).

### Data Collection Process

We created a data extraction form to assemble previously defined relevant information from the studies: country, study design, dates of enrollment, population, penicillin regimen, sample size and adverse reactions. One author extracted the data (TFG) and another (MTS) confirmed the extracted information. We contacted studies’ corresponding authors to obtain any additional data not stated in the reports. We labeled the study as “confirmed multiple exposure” if all the patients of the study received more than one dose of penicillin.

### Risk of Bias and Quality of Evidence Assessment

To assess the risk of bias in the randomized clinical trials, we considered the Cochrane Collaboration’s tool [23]. For observational studies we assessed: eligibility criteria, measurements of exposures and outcomes, control of confounding, and follow-up [24].

We used the Grading of Recommendations Assessment, Development and Evaluation (GRADE) approach to assess the quality of the evidence [25]. This method rates the quality of evidence as high, moderate, low or very low. We ranked the outcomes as critical, important or not important and then assessed study limitations (risk of bias), inconsistency, indirectness of evidence, imprecision, publication bias, and factors that could increase the quality of the evidence. The quality assessment was considered when interpreting the findings.

### Data Analysis

The primary outcome measured was the incidence of serious adverse reactions in pregnant women due to treatment with benzathine penicillin for preventing congenital syphilis. As serious adverse reactions we considered anaphylaxis and death, but we did not summarize them as a composite outcome.

Absolute risks from individual studies were recalculated and presented along with 95% confidence intervals (95% CI) by the Mid-P test [26]. From the resulting confidence intervals, we did meta-analyses by random effects model using the method of DerSimonian and Laird [27]. As the incidence was small, we adjusted the values with exponential and natural logarithm function [28]. We did all the analysis on STATA (v. 10.1) statistical package. Chi^2^, I^2^ and Tau^2^ tests were calculated to estimate heterogeneity. We performed sensitivity analysis to identify the potential sources of heterogeneity across studies.

## Results

### Study Selection

**Figure 1:**
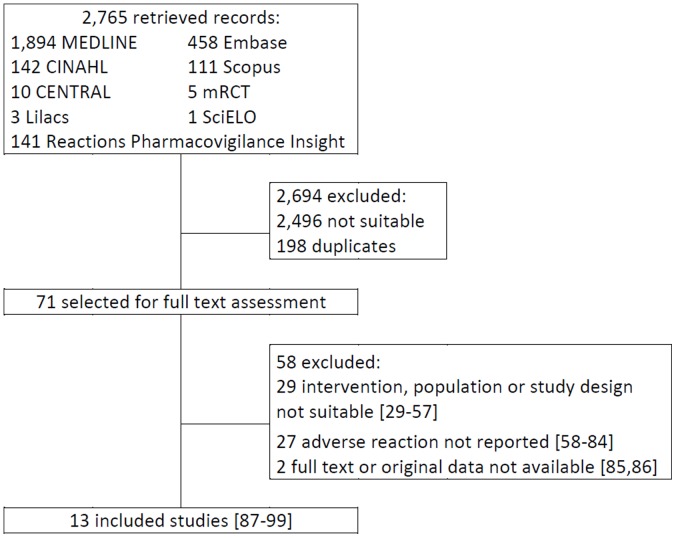
Results of search, selection and inclusion of studies in the review.

Our literature search identified 2,765 articles (Figure 1). After screening the titles and abstracts, 71 were selected for full text assessment [29]–[99], and 13 were included in the review (N = 3,466,780 patients) [87]–[99].

### Study Characteristics

We could not identify any studies whose primary objective was to measure the incidence of adverse reactions in pregnant women with syphilis treated with benzathine penicillin. Studies that included pregnant women aimed to assess the effectiveness of benzathine penicillin for preventing congenital syphilis, and the incidence of adverse reactions was recorded as a secondary endpoint. All studies included in our review had a cohort design, either prospective or retrospective. The benzathine penicillin regimen varied from 1 to 4 doses of 1.2 million international units (MIU) and its use was compared to no treatment or erythromycin, or no comparison was done. Table 1 depicts the main characteristics of the included studies.

**Table 1:** Main characteristics of included studies.

Study	Studydesign	Dates of enrolment	Country	Population(diagnosis method if available)	Benzathinepenicillin regimen	Control group
Shafer 1954 [87]	prospective cohort	1950–1952	USA	adults with sexually transmitted diseases	1 or 2 doses of 2.4 MIU, IM*	no control group
Smith 1956 [88]	prospective cohort	1946–1950	USA	adults with syphilis or gonorrhea	1, 2 or 3 doses of 2.4 MIU, IM	no control group
Willcox 1957 [89]	prospective cohort	1946–1956	USA	adults with syphilis or gonorrhea	1 to 4 doses of 2.4 MIU, IM	no control group
Hsu 1958 [90]	prospective cohort	several years	USA	adults with rheumatic fever	1.2 MIU, IM every fourweeks for several years	no control group
Phaosavasdi 1989 [91]	prospective cohort	1984–1985	Thailand	pregnant women with positive treponemal serological test (RPR, VDRL, TPHA)	1, 2 or 3 doses of 2.4 MIU, IM	erythromycin 2 g dailyper 30 days, orally
International rheumatic fever group 1991 [92]	prospective cohort	1988–1990	11 countries†	adults and children with rheumatic fever	1.2 MIU, IM every 4weeks for three years	no control group
Jenniskens 1995 [93]	prospective cohort	1992–1993	Kenya	pregnant women with positivetreponemal serological test (RPR)	2.4 MIU, IM	no treatment
Napoli 2000 [94]	prospective cohort	1999–2000	USA	adults with streptococcal pharyngitisprophylaxis	1.2 MIU, IM	no control group
Apter 2004 [95]	prospective cohort	1987–2001	UK	adults and children who received penicillin prescription	1 or 2 prescriptions of penicillin*	no control group
Watson-Jones 2005 [96]	retrospective cohort	2000–2001	Tanzania	pregnant women with positive treponemalserological test (RPR, TPHA, FTA-ABS)	1 dose of 2.4 MIU, IM	no treatment
Bronzan 2007 [97]	prospective cohort	2001–2002	South Africa	pregnant women with primary, secondary,or early latent syphilis (RPR, TPHA)	1, 2 or 3 doses of 2.4 MIU, IM	no treatment
Carles 2008 [98]	retrospective cohort	1992–2004	French Guiana	pregnant women with positive treponemalserological test (VDRL, TPHA)	1, 2 or 3 doses of 2.4 MIU, IM	no treatment
Li 2012 [99]	retrospective cohort	2001–2008	China	adults with sexually transmitted diseases	3 doses of 2.4 MIU, IM	erythromycin 2 g daily per14 days, orally ordoxycycline 200 mg orallyfor 15 days

Notes:

* It may have been included also patients treated with other types of penicillin.

† Argentina, Chile, China, India, Jamaica, Korea, Kuwait, New Zealand, Taiwan, Thailand, Venezuela.

Abbreviations:

MIU: mega internacional units.

IM: intramuscular.

RPR: Rapid Plasma Reagin.

VDRL: Veneral Disease Research Laboratory.

TPHA: Treponema Pallidum Hemagglutination.

MHA-TP: microhemagglutination *T. pallidum.*

FTA-ABS: test or fluorescent treponemal antibody–absorption.

### Quality of the Evidence

The quality of evidence of all outcomes was considered very low (Table 2). The quality was rated down from low, because of the observational design of the studies, to very low, due to limitations (mainly on the measurement of outcome, as the studies were not designed to report the incidence of adverse reactions in pregnant women), inconsistency (high heterogeneity), and indirectness (population different from pregnant woman with syphilis).

**Table 2:** Quality of evidence profile for the assessed outcomes, adapted from GRADE [25].

Outcome (population)	Quality assessment					Quality	Importance
	N. studies	Limitation	Inconsistency	Indirectness	Imprecision		
death (pregnant women)	five [91], [93], [96]–[98]	serious *	−	−		very low	Critical
death (general population)	eight [87]–[90], [92], [94], [95], [99]	serious†	no important inconsistency	very serious§	no important imprecision	very low	Critical
anaphylaxis (pregnant women)	five [91], [93], [96]–[98]	serious*	−	−		very low	Critical
anaphylaxis(general population)	eight [87]–[90], [92], [94], [95], [99]	serious†	no important inconsistency	very serious§	no important imprecision	very low	Critical
incidence of adverse reactions(pregnant women)	five [91], [93], [96]–[98]	serious*	−	−		very low	Important
incidence of adverse reactions(general population)	eight [87]–[90], [92], [94], [95], [99]	serious†	very serious‡	very serious§	no important imprecision	very low	Important

Notes:

All studies had observational design.

Publication bias could not be objectively assessed due to the small numbers of studies.

Imprecision could not be assessed since adequate meta-analysis calculation could not be performed.

RR: relative risk.

NE: Non-estimable.

−We could not access the item for this outcome.

* Flawed measurement of outcome, as the study did not aimed to report the incidence of adverse reaction in pregnant women.

† Some studies did not report the incidence of adverse reaction; the data was obtained with the authors.

‡ Heterogeneous results across studies were observed.

§ Different population (patients were not pregnant women and did not have syphilis) and intervention. In one study [95] other types of penicillin, besides benzathine, may have been used.

### Absolute Risk from Individual Studies and Pooled Results

Studies did not report any case of anaphylaxis or death in pregnant women treated with benzathine penicillin. The incidence of adverse reactions in this population is presented on Table 3. In total 1,244 women were assessed and only one case of skin rash was reported. As all studies had null events, a meta-analysis was not possible.

**Table 3:** Incidence of adverse reactions in pregnant women treated with benzathine penicillin for preventing congenital syphilis, 1954–2012.

Study	Penicillin treatment group		No penicillin treatment group	
	No. of patients	Events	No. of patients	Events
Phaosavasdi 1989 [91]	191	1*	6	0
Jenniskens 1995 [93]	751	0†	109	0†
Watson-Jones 2005 [96]	88	0†	56	0†
Bronzan 2007 [97]	141	0†	31	0†
Carles 2008 [98]	73	0† ‡	12	0†

Notes:

* Skin rash.

† Data obtained from contact with corresponding author.

‡ All patients received dexamethasone injection.

In studies whose primary objective was to evaluate the incidence of adverse reactions from benzathine penicillin treatment, no pregnant women were included. From 2,028,982 patients treated with benzathine penicillin, 4 died from an adverse reaction (Table 4). The absolute risk of individual studies ranged from 0% (95% CI: 0%–0.274%) to 3.125% (95% CI: 0.156%–14.460%) and the pooled risk of death was zero (I^2^ = 0%).

**Table 4:** Incidence of anaphylaxis or death in general population treated with benzathine penicillin: individual and pooled results, 1954–2012.

Individual studies orpooled results	No. ofpatients	Death		Anaphylaxis	
		Events	Absolute risk (95% CI)	Events	Absolute risk % (95% CI)
Shafer 1954 [87]	70,037	2	0.003 (0.001–0.009)	0	0 (0–0.004)
Smith 1956 [88]	7,109	0	0 (0–0.042)	0	0 (0–0.042)
Willcox 1957 [89]	895	0	0 (0–0.334)	0	0 (0–0.334)
Hsu 1958 [90]	32	1	3.125 (0.156–14.460)	1	3.125 (0.156–14.460)
International rheumatic fevergroup 1991 [92]	1,790	1	0.126 (0.006–0.623)	4	0.223 (0.071–0.538)
Napoli 2000 [94]	9,203	0	0 (0–0,032)	2	0.022 (0.004–0.072)
Apter 2004 [95]	2,017,957	0	0 (0–0,001)	47*	0.001 (0,002–0,003)
Li 2012 [99]	1,094	0‡	0 (0–0.274)	0‡	0 (0–0.274)
Pooled result	2,108,117	4	0 (0–0); I^2^ = 0%	54	0.002 (0–0.003); I^2^ = 12%

Notes:

* 16 patients had anaphylaxis after the first prescription of penicillin and 32 had anaphylaxis after the second prescription of penicillin. One patient had anaphylaxis in both prescriptions. Total patients that experienced anaphylaxis in this study = 47.

‡ There were reported 16 events of Jarisch-Herxheimer reaction, which were not considered adverse drug reaction in present review.

Fifty four patients of 2,028,982 treated with penicillin suffered anaphylaxis. Across studies, the absolute risk ranged from 0% (95% CI: 0%–0.004%) to 3.125% (95% CI: 0.156%–14.460%). Studies that included patients with multiple exposures to penicillin had a higher incidence of anaphylaxis [90], [92]. The pooled risk from the meta-analysis was 0.002% (95% CI: 0%–0.003%; I^2^ = 12%); from this estimate we can expect 0 to 3 cases of anaphylaxis per 100,000 treated patients.

For any adverse reaction 6,377 cases were observed among 3,465,322 treated patients (Table 5); no pregnant women were assessed. The absolute risk ranged from 0% (95% CI: 0%–0.274%) to 18.750% (95% CI: 7.968%–34.980%) among studies. The risk was higher in studies that included patients with confirmed multiple exposures to penicillin [90], [92], [95], [99], when compared with the ones in which the patients received only one dose of penicillin or multiple exposure to penicillin was not confirmed [87]–[89], [94], [95].

**Table 5:** Incidence of adverse reactions in general population treated with benzathine penicillin: individual and pooled results, 1954–2012.

Individual studies orpooled results	No. ofpatients	Events	Absolute risk % (95% CI)	Type of adverse reaction(n of patients, if available)
Shafer 1954 [87]	70,037	56*	0.080 (0.061–0.103)*	only severe adverse events reported; type not available
Smith 1956 [88]	7,109	18	0.253 (0.155–0.392)	urticaria, nausea and vomiting
Willcox 1957 [89]	895	26†	2.905 (1.947–4.168)	urticaria, urticaria, edema, asthma, rash, dyspnea, tetany, faintness, dizziness, diarrhea, urticaria, vomiting
Hsu 1958 [90]	32	6	18.750 (7.968–34.980)	anaphylaxis (1), edema of lips, pruritic eruption (3), serum sickness (2)
International rheumatic fever group 1991 [92]	1,790	57	3.184 (2.443–4.077)	pruritus or urticaria (33), macopapular rashes (11), arthralgia (8), anaphylaxis (4), wheeze (1)
Napoli 2000 [94]	9,203	2*	0.022 (0.004–0.072)*	anaphylaxis (2)
Apter 2004 [95] – one prescription of penicillin	3,375,162	6,212	0.184 (0.179–0.189)	allergic-like event: adverse drug reaction, anaphylaxis, angioedema, erythema multiforme, toxic epidermal necrolysis, urticaria
Apter 2004 [95] – two prescriptions of penicillin within 60 days	2,017,957‡	3,509‡	0.174 (0.168–0.180)	
Li 2012 [99]	1094	0§	0 (0–0.274)	None||
Pooled result¶	3,465,322	6,377	0.169 (0.073–0.265); I^2^ = 97%	−

Notes:

* Only severe adverse reaction was reported.

† Probable cases.

‡ These cases are included in the previous data (one penicillin prescription).

§ Data obtained from contact with corresponding author.

|| The study reported 16 events of Jarisch-Herxheimer reaction, but we did not consider them as adverse events.

¶ For the polled result only the incidence of adverse reaction with one prescription of penicillin was considered for Apter 2004 [95] study.

The pooled risk for any adverse reaction was 0.169% (0.073%–0.265%; I^2^ = 97%). The statistical heterogeneity was very high. In the sensitivity analysis we investigated the effect of older studies, the level of country economic development where the studies were conducted, the stage of disease, and the dosing regimens. It is clear that studies were performed in different decades and settings, and this may be the main causes of the heterogeneity we found, but we could not identify the statistical sources of heterogeneity, nor could we derive more homogeneous results from the sensitivity analysis.

## Discussion

Our findings show that the incidence of serious adverse reactions to benzathine penicillin in pregnant women was very low: no severe or fatal cases were reported. In the general population, a study population with a much larger sample size, the risk of serious adverse reactions was also small, and the pooled risk of death due to penicillin treatment was virtually zero. We rated the quality of the evidence as very low, resulting in a classification of the final evidence as inconclusive. Studies that assessed benzathine penicillin effectiveness in pregnant women did not plan the sample size to measure adverse reactions, therefore the statistical power to detect low frequency adverse reactions was suboptimal [100]. Further well-designed studies may change or confirm the findings [25].

Patients with multiple exposures to benzathine penicillin had a higher incidence of adverse reactions. That finding correlates with clinical data in which patients with more frequent exposure to penicillin have a higher chance of experiencing adverse reactions [101].

Most of the studies had no events of serious adverse reactions, which hampered the summarization of the risk. To conduct a meta-analysis, we tried different approaches, like replacing the zero events with 0.5 [102], and excluding the studies without events from the analysis. Such attempts overestimated the risk and we considered those results inadequate. The method of DerSimonian and Laird [27], calculated from confidence intervals of individual studies, was the best model to estimate the risk and was selected to estimate the pooled risk. Even using that method, in situations with very low rates like the risk of death in the general population, the pooled estimate resulted in “zero” risk. The risk does exist; studies reported four events in more than two million patients. But the meta-analysis – weighting the events by each study population – resulted in rounding the result to zero.

The included studies were performed in different decades, and disparate benzathine penicillin regimens and stages of disease were assessed. It is possible that the diagnosis of adverse reaction varied across studies, as well as the benzathine penicillin preparation and adjunct components, with greater variation in the older studies. A possible measurement bias may derive from study design, but this factor could not be investigated due to the low number of retrospective as compared with prospective studies. Such clinical and methodological diversity might explain the heterogeneity observed on the outcome of any adverse reaction and raises concerns about the external validity of the data [23], [28].

Given the widely documented effectiveness of benzathine penicillin in preventing adverse pregnancy outcomes in mothers with syphilis such as stillbirth, preterm delivery, perinatal death and congenital syphilis [5]–[9], limiting access to benzathine penicillin due to concerns about adverse penicillin reactions cannot be justified. However, in all settings in which benzathine penicillin is administered, it is critical that providers have adequate training and resources to provide sterile injections and management of anaphylaxis. A component of such training should include the routine assessment of a history of allergic reactions to antibiotics including penicillin. A history of penicillin allergy seems a good screening measure to predict the likelihood of a serious reaction. Those with a positive history of penicillin allergy might still benefit from targeted hypersensitivity testing to exclude a true allergy, if available [18].

Even though the risk of death and serious adverse reactions due to benzathine penicillin seem to be very low, research is needed to specifically determine and monitor the incidence of adverse reactions in pregnant women, including a more clear understanding of the rates of mild, moderate and severe adverse reactions. Large medical databases, for example, could provide a better estimate of the incidence of adverse reactions in the population [103], [104]. Furthermore, countries should consider actively monitoring the frequency of adverse events due to benzathine penicillin administration in pregnant women through pharmacovigilance systems [105].

It is also reasonable that researchers include the incidence and type of adverse reaction as one of the outcomes when designing prospective studies of benzathine penicillin use in pregnancy. In our literature search we found many studies that could have added more information to the present evidence base if the incidence and type of adverse reaction had been systematically recorded [58]–[61], [67]–[75], [80]–[82].

### Conclusion

No case of serious adverse reactions to benzathine penicillin in pregnant women was reported and in the general population the incidence was very low. For clinical practice and public health policy our findings suggest that the risk of adverse reactions does not outweigh the benefits of benzathine penicillin use for maternal syphilis treatment and congenital syphilis prevention. Health authorities should eliminate any policy barriers to the administration of benzathine penicillin. Future studies about penicillin use should record the incidence and type of adverse reactions during pregnancy. Such research efforts are likely to strengthen the available evidence.

## Supporting Information

Table S1 Search strategy for MEDLINE (via PubMed).
(DOCX)
