# Supplementary material for: Safety of Benzathine Penicillin for Preventing Congenital Syphilis: A Systematic Review
Source: PLoS One. 2013 Feb 21;8(2):e56463. doi: 10.1371/journal.pone.0056463 (PMC3578834; doi:10.1371/journal.pone.0056463)
Supplement: Table S1 — Search strategy for MEDLINE (via PubMed). (DOCX) [file pone.0056463.s001.docx]

Table S1. Search strategy for MEDLINE (via PubMed)

| Step | Search terms |
| --- | --- |
| #1 | “pregnancy”[mesh] OR “pregnant women”[mesh] OR “women”[tiab] OR “pregnant”[tiab] OR “woman”[tiab] OR “pregnancies”[tiab] OR “gestation”[tiab] OR “pregnancy”[tiab] |
| #2 | “syphilis”[mesh] or “syphilis, congenital”[mesh] or “syphilis”[tiab] or “syphilis, congenital”[mesh] or “congenital syphilis”[tiab] or “great pox”[tiab] or “lues”[tiab] or “chancre”[mesh] or “chancre”[tiab] or “treponemal infections”[mesh] or “treponemal infections”[tiab] or “bejel”[tiab] or “treponema”[tiab] |
| #3 | “penicillin g”[mesh] OR “penicillin g benzathine”[mesh] OR “penicillin”[tiab] OR “benzylpenicillin”[tiab] OR “benzathine”[tiab] OR “bicillin”[tiab] |
| #4 | “prevention and control”[subheading] or “preventive therapy”[tiab] or “prophylaxis”[tiab] or “preventive”[tiab] or “prevention”[tiab] or “therapy”[subheading] or “treatment”[tiab] or “disease management”[tiab] |
| #5 | “adverse reaction”[tw] OR “side effect”[tw] OR “adverse event”[tw] OR “allergy”[tiab] OR “allergic”[tiab] |
| #6 | randomized controlled trial[pt] OR (randomized[tiab] AND controlled[tiab] AND trial[tiab])) OR “cohort studies”[mesh] OR “Epidemiologic Studies”[mesh] OR “Data Collection”[mesh] OR “longitudinal studies”[Mesh] OR /incidence |
| #7 | #1 AND #2 AND (#3 OR #4) |
| #8 | #3 AND #5 |
| #9 | (#7 OR #8) AND #6 |
